# Oral Ketone *β*-Hydroxybutyrate Supplement Retards the Loss of GFR in Alport Mice on Dual Renin-Angiotensin System/Sodium-Glucose Transporter 2 Blockade

**DOI:** 10.34067/KID.0000000747

**Published:** 2025-03-11

**Authors:** Linus P. Schreier, Zhihui Zhu, Yoshihiro Kusunoki, Chenyu Li, John Ku, Martin Klaus, Hans-Joachim Anders

**Affiliations:** 1 Division of Nephrology, Department of Medicine IV, Hospital of the Ludwig-Maximilians-University, Munich, Germany; 2 Department of Cardiac Surgery, Beijing Anzhen Hospital, Capital Medical University, Beijing, China; 3 Department of Rheumatology, Endocrinology, and Nephrology, Faculty of Medicine and Graduate School of Medicine, Hokkaido University, Sapporo, Japan

**Keywords:** CKD, ESKD, fibrosis, genetic kidney disease, GFR, glomerulopathy

## Abstract

**Key Points:**

- There is an unmet medical need for testing novel CKD substances to create more effective combination therapies.
- In our CKD mouse model, ketone supplementation retards the loss of GFR beyond the current standard of care, but no effect on lifespan was observed.
- Ketone supplementation suppresses kidney inflammation and fibrosis.

**Background:**

Several studies suggest that dietary *β*-hydroxybutyrate (BHB) supplementation delays the progression of CKD by suppressing inflammation and fibrosis. We hypothesized that the oral supplementation with the BHB precursor 1,3-butanediol in addition to inhibitors of the renin-angiotensin system (RAS) and sodium-glucose transporter 2 (SGLT2) would be superior to dual RAS/SGLT2 blockade alone in attenuating the loss of GFR in Col4a3-deficient mice with Alport nephropathy, a spontaneous model of progressive CKD.

**Methods:**

We performed a negative-controlled study in Col4a3-deficient mice with Alport nephropathy. Treatment was initiated at a late stage of the disease at the age of 6 weeks. Mice were fed food admixes of 10 *μ*g/g ramipril plus 30 *μ*g/g empagliflozin with or without addition of 0.04 g/g 1,3-butanediol (concentration per gram of bodyweight). The mice were monitored daily and sacrificed on reaching renal failure. The GFR was measured at the start of the treatment and after 1 and 4 weeks.

**Results:**

The addition of BHB significantly attenuated the loss of GFR beyond the effect of dual RAS/SGLT2 blockade. The mean GFR after 4 weeks of treatment was 0 ± 0 *μ*l/min (vehicle), 57 ± 54 *μ*l/min (renin-angiotensin system inhibitors + sodium-glucose transporter 2 inhibitors), and 139 ± 69 *μ*l/min (renin-angiotensin system inhibitors + sodium-glucose transporter 2 inhibitors+1,3-butanediol). No additional effects on lifespan could be observed. Kidney RNA sequencing revealed significant protective effects on inflammation when adding the BHB precursor 1,3-butanediol to RAS/SGLT2 inhibition. In histopathology, antifibrotic effects were seen on BHB addition.

**Conclusions:**

The results in mice suggest that BHB supplementation improves the GFR in Alport syndrome by suppressing inflammation and fibrosis. However, the effects did not lead to a significant increase in lifespan. Furthermore, the observed effects stay behind the effects of finerenone as a combination partner, which was tested earlier in the same mouse model.

## Visual Abstract

**Figure:**
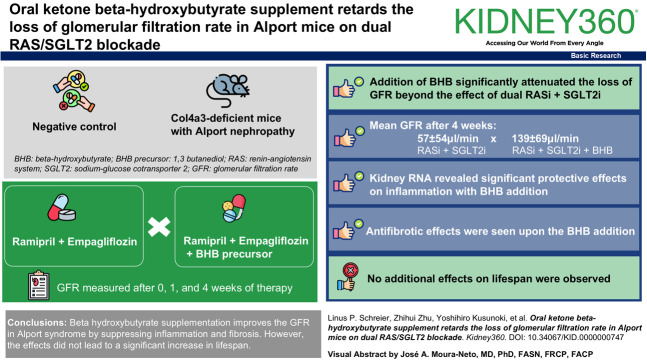

## Introduction

In patients with CKD, dual inhibition of the renin-angiotensin system (RAS) and sodium-glucose transporter 2 (SGLT2) sustains the decline of GFR more potently than RAS inhibition alone.^1^ Adding further renoprotective drugs can elicit synergistic renoprotection in CKD patients with diabetes, but this is unclear for patients with nondiabetic CKD.^2–4^ SGLT2 inhibitors and other drugs reduce metabolic stress, namely, in tubular epithelial cells; hence, targeting kidney metabolism is attractive for therapeutic intervention.^5^ For example, a ketogenic diet is believed to elicit renoprotective effects by modulating kidney metabolism and suppressing residual inflammation and fibrosis, but long-term adherence to such diets is difficult.^6,7^ Several studies suggest that dietary supplementation with the ketone *β*-hydroxybutyrate (BHB) can have similar protective effects by inducing ketosis.^8–11^ For example, BHB can reduce oxidative tissue stress and increase global histone acetylation, probably by inhibiting endogenous histone deacetylases.^9^ BHB inhibits the NOD-like receptor family pyrin domain-containing 3 (NLRP3) inflammasome^12^ and reduces GFR loss and kidney fibrosis by shifting the phenotype of intrarenal macrophages toward anti-inflammatory macrophages.^11^ However, if BHB supplementation has beneficial effects beyond standard of care is unknown. We hypothesized that oral BHB supplementation added to dual RAS/SGLT2 inhibition could attenuate the progressive loss of GFR.

## Methods

### Animals and Study Design

Col4a3−/− (Col4a3tm1Dec) mice with a 129/SvJ background (Jackson Lab, Bar Harbor, ME) are a widely used animal model of progressive CKD in Alport syndrome^13^ and allow study of the spontaneous progression of CKD up to the stage or uremia. Col4a3−/− mice were bred under specific pathogen-free housing conditions, cohoused with enrichment in a 12-hour light and dark cycle with free access to chow and autoclaved water and genotyped, as described earlier.^14,15^ All experimental procedures were approved by the local government authorities (approval code ROB-55.2Vet-2532.Vet_02-20-233) according to the European equivalent of the National Institutes of Health's Guide for the Care and Use of Laboratory Animals (directive 2010/63/European Union). Inclusion and exclusion criteria for participant mice are reported in the provided Supplemental File. Mice were enrolled between February 2022 and September 2023. Mice were fed a food admix of either (*1*) vehicle, (*2*) 10 *μ*g/g ramipril plus 30 *μ*g/g empagliflozin, or (*3*) 10 *μ*g/g ramipril plus 30 *μ*g/g empagliflozin plus 0.04 g/g 1,3-butanediol (the drug concentration is given per gram of bodyweight). The different types of food preparations were marked by different food colors. Treatment started at age 6 weeks for 8 weeks. For the cross-sectional analysis, a subgroup of mice in every group was treated for 2.5 weeks and euthanized for blood sampling and kidney collection at age 8.5 weeks. During the treatment, all animals underwent measurements of their GFR. All animals were sacrificed by cervical dislocation under isoflurane anesthesia. The prespecified primary end point was mean overall survival. Primary and secondary end points were the measurement of GFR, proteinuria, serum BUN, potassium, phosphate, urine glucose, histology, immunostaining, quantitative PCR (qPCR), and RNA-sequencing. A study schematic can be found in the provided supplement (Supplemental File 1).

### Survival Study

During the experiment, mice were scored daily to meet animal welfare criteria and to terminate animals before suffering from uremia. After reaching predefined termination criteria (Supplemental File 2), the mice were euthanized and organs, blood, and urine were obtained. The time point of termination was defined as death by uremia. All parameters of the study were predefined and not changed later.

### Randomization, Blinding, Grouping, and Sample Size

Mice received either (*1*) vehicle, (2) 10 *μ*g/g ramipril plus 30 *μ*g/g empagliflozin, or (*3*) 10 *μ*g/g ramipril plus 30 *μ*g/g empagliflozin plus 0.04 g/g 1,3-butanediol. The experiment was approved by the local government authorities (approval code. ROB-55.2-2532.Vet_02-20-223). Mice were assigned to the different treatment groups successively; there was no blinding. Confounding was limited by an equal sex distribution within groups, as well as by the restriction to the administered special food. The renin-angiotensin system inhibitors + sodium-glucose transporter 2 inhibitors (RASi + SGLT2i)+1,3-butanediol group included 20 mice (50% female and 50% male): 16 mice were used in the lifespan study and four mice in the 8.5 weeks study. For the other groups, 20 mice (50% female and 50% male) were used in the lifespan study. The comparison for the 8.5 weeks study included four mice in every group.

### Sample Size Calculation

Group comparisons are determined about the (kidney) survival time, defined as achieving a stress score >2 for more than 6 hours (Supplemental File 2). A comparison is made with RASi+SGLT2i-treated animals. A minimum survival time of 70 days in RAS+SGLT2i was estimated. The median duration in the RASi+SGLT2i was estimated to be 80 days. An extension to 95 days was set to be clinically relevant. It can be assumed that there will be no failures up to day 70 as this is the estimated minimum lifespan of RAS+SGLT2i-treated mice. This estimation was proven to be right later. The log-rank test is used as a statistical test. This corresponds to a clinically relevant hazard coefficient of (95–70)/(80–70)=2.5. With an error of the first type of *α*=0.05 and an error of the second type of *β*=0.3, the sample size is *n*=16 per group. The use of a two-sided test is necessary because it cannot be ruled out that 1,3-butanediol leads to a shortening of the (kidney) survival time.

### Measurement of GFR

GFR was measured in conscious and unrestricted mice before the onset of treatment and two subsequent time points at 1 and 4 weeks after the initiation of treatment. A transcutaneous detector system for FITC-Sinistrin clearance kinetics (Mannheim Pharma & Diagnostics GmbH, Germany) was used as described.^16,17^

### Measurement of Serum BUN, BHB Levels, Potassium, Phosphate, and Urine Glucose

Urine samples were collected every week by applying gentle pressure on the mice's bladder for the estimation of creatinine (DiaSys Diagnostic Systems, Holzheim, Germany) and albumin (Bethyl Laboratories, Montgomery). The levels of BUN in mouse serum were measured using the enzymatic method (Urea FS, Diasys, Holzheim, Germany). BHB levels were measured using a BHB ELISA kit (Sigma-Aldrich, MO). Urinary glucose was measured using the hexokinase/glucose-6-phosphate dehydrogenase method (Cayman Chemical, MI). The urinary glucose-to-creatinine ratio was calculated as (mg/mg)=urine glucose (mg/dl)/urine creatinine (mg/dl). All ELISA and colorimetric assays followed the manufacturer's protocols. Potassium and phosphate were tested in SYNLAB Munich.

### Histology, Immunostaining, and Quantitative Analysis

Transverse kidney sections were sectioned at 3 *μ*m and stained with periodic acid–Schiff (PAS), Picro-Sirius red, alpha smooth muscle actin (*α*SMA), p57 (Supplemental File 3), and F4/80 according to standard protocols. Images of the sections were scanned by light microscopy, and digitalized, quantitative analysis was performed by the software of QuPath. The pathologic scoring for kidney injury (from PAS-stained whole kidney slides) was based on (*1*) tubular necrosis, (*2*) tubular dilation, (*3*) tubular cast, (*4*) loss of brush border, and (*5*) interstitial edema. In each category, scores from 1 to 10 were given, representing 10%–100% occurrence of the mentioned criteria. The overall score was calculated by dividing the sum of the scores by the number of categories. The pathologic scoring of tubular injury was based on the same scoring system, but did not include the criteria of (*4*) loss of brush border and (*5*) interstitial edema. For the Sirius Red staining, kidney fibrosis was quantified by evaluating the percentage of collagen (red) in whole kidney slides. After Pico Sirius red staining, the sections were digitized and the whole kidney was annotated in QuPath.^18^ RGB image sections were converted to the hue, saturation, value color model using the NumPy program.^19^ Pixels with the values (11/12<c1 or <1/12, c2>0,1, c3>0,1) were interpreted as fibrosis. Ultimately, thresholds were obtained for the entire kidney section, allowing the proportion of fibrosed area in the section to be calculated. For the *α*SMA staining, sections were stained with rabbit anti-mouse *α*SMA antibody by an immunohistochemistry method (1:500, Dako, Hamburg, Germany). QuPath software was used to calculate the percentages of smooth muscle actin–positive nuclei per kidney by setting a threshold for fibrosis (th.150; average channels). For the F4/80 staining, sections were stained with antibody (CI-A3-A) by an immunohistochemistry method (1:100, Novusbio NB600-404). Whole kidney slides were annotated in QuPath, and the F4/80 positive area of the whole kidney was calculated using a threshold (th.130; average channels).

### RNA-Sequencing

RNA was extracted from the kidneys of four *Col4a3*−/− mice per group at age 8.5 weeks. The library construction and sequencing were performed by Beijing Genomics Institute using a DNBSEQ (G400) platform. The RNAs were subjected to 100-bp paired-end sequencing. The bioinformatics workflow, which included data filtering, mapped transcript prediction, analysis of differential gene expression, and gene ontology, was performed in accordance with the protocols of HISAT2,^20^ SAMtools,^21^ featureCounts,^22^ eisaR,^23^ and clusterProfiler.^24^ The RNAseq data are available in the Gene Expression Omnibus database (GSE226353) and (GSE287708).

### RT-qPCR

Gene expression analysis via reverse transcription qPCR (RT-qPCR) was carried out in the following way: total RNA was isolated using an RNA extraction kit (Life Technologies, Darmstadt, Germany) according to the manufacturer's instructions, and RNA quality was assessed using nano drop technology. After the isolation of RNA, cDNA was generated using reverse transcriptase (Superscript II; Invitrogen, Carlsbad). SYBR Green Dye detection system was used for quantitative real-time PCR on Light Cycler 480 (Roche, Mannheim, Germany) using 18s ribosomal RNA as a housekeeping gene. Gene-specific primers blasted with basic local alignment search tool by the ensembl project and National Center for Biotechnology Information primer-basic local alignment search tool (Metabion, Martinsried, Germany) were used, as listed in Supplemental File 4. Nontemplate controls consisting of all used reagents were negative for target and housekeeping genes. To reduce the risk of false-positive crossing points, the high confidence algorithm was used. The melting curve profiles were analyzed for every sample to detect eventual unspecific products or primer dimers, as a housekeeping gene 18s RNA was taken.

### Statistical Analysis

Data are presented as mean with SD or as boxplot statistics. Before statistical analysis, data were analyzed for normal distribution by checking the distribution of the data against the expected normal distribution with a quantile-quantile plot and confirmation with the Shapiro-Wilk test. We tested normally distributed data for statistically significant differences using ANOVA, and *post hoc* Tukey correction was used for multiple comparisons. Non-normally distributed data were compared using Kruskal-Wallis testing with the *post hoc* Dunn test for multiple comparisons. When only comparing all groups to one group, the Dunnett multiple comparison test was used for normally distributed data and the Dunn multiple comparison test was used for non-normally distributed data. When two groups were compared, either an unpaired *t* test was used for normally distributed data or a Mann–Whitney test was used for non-normally distributed data. Survival was plotted on Kaplan-Meier curves, and comparisons between groups were evaluated using log-rank tests. GFR decline was tested for significance using two-way ANOVA followed by a Turkey multiple comparison test for the time points 7 and 10 weeks. All statistical analyses were performed with R (3.5.3) or GraphPad Prism 9.0.

## Results

**Figure 1:**
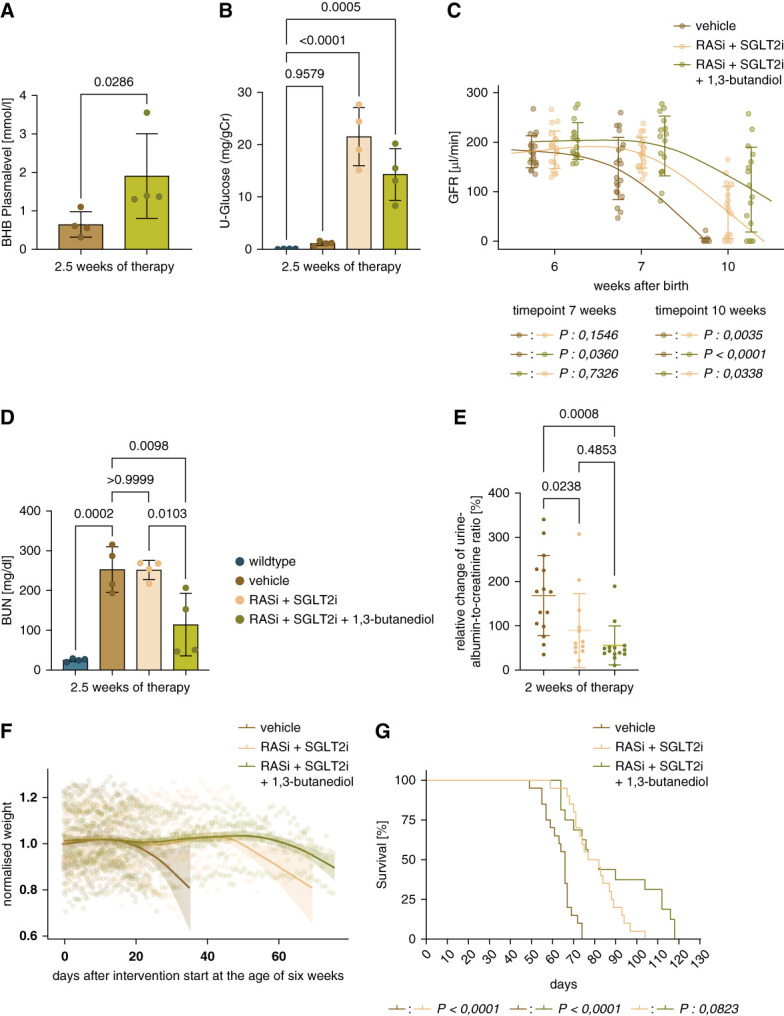
**GFR curve, markers of kidney function, weight loss curve, BHB serum levels, glucosuria, and overall survival.** (A) BHB plasma levels at 2.5 weeks of therapy in vehicle and RASi/SGLT2i/1,3-butanediol groups. (B) Urine glucose levels for vehicle, RASi+SGLT2i, and RASi+SGLT2i+1,3-butanediol group. (C) Effects of vehicle, RASi+SGLT2i, and RASi+SGLT2i+1,3-butanediol therapy on GFR decline. (D) BUN levels after 2.5 weeks of therapy for vehicle, RASi+SGLT2i, and RASi+SGLT2i+1,3-butanediol groups. (E) Relative change in urine albumin-to-creatinine ratio levels in percentage within 2 weeks of therapy for vehicle, RASi+SGLT2i, and RASi+SGLT2i+1,3-butanediol groups. (F) Change of bodyweight after therapy start in different groups (shown as a percentage of initial body weight). (G) Survival rate (uremia-free survival) of vehicle, RASi+SGLT2i, and RASi+SGLT2i+1,3-butanediol groups. Testing for significance was performed using a Mann–Whitney test in (A) and (B) using an ordinary one-way ANOVA followed by a Dunnett multiple comparisons test. For (C), a two-way ANOVA with the following Turkey multiple comparison test was used for the time points 7 and 10 weeks. For (D and E) through ordinary one-way ANOVA with the following Turkey multiple comparison test. For the comparison of the survival curves in (G), a Kaplan-Meier analysis was used. All quantitative data are means±SD. *P* value of <0.05 was considered to indicate statistical significance. BHB, *β*-hydroxybutyrate; RASi, renin-angiotensin system inhibitors; SGLT2i, sodium-glucose transporter 2 inhibitors; U-Glucose, urine glucose.

Up to the age of 6 weeks, the time of randomization, *Col4a3*−/− mice developed progressive Alport nephropathy with no significant differences between groups in body weight and GFR (Table 1). On average, GFR (*µ*L/min) was 181±32 in vehicle, 185±38 in RASi+SGLT2i, and 202±37 in RASi+SGLT2i+1,3-butanediol group. In addition, there were no sex-specific differences between groups regarding body weight and GFR at the start point of intervention (Supplemental Tables 1 and 2). As we have shown earlier, Alport nephropathy in 6-week-old *Col4a3*−/− mice is evident by considerable glomerulosclerosis, tubular atrophy, kidney injury, and *α*SMA+/Sirius Red+ interstitial fibrosis.^25^ Thus, we initiated treatments at a clinically relevant stage of CKD. Sufficient exposure to 1,3-butanediol and empagliflozin was confirmed by measuring BHB levels in the blood and glucose levels in the urine after 2.5 weeks of therapy (Figure 1, A and B). Consistent with previous studies,^25^ GFR rapidly declined in untreated *Col4a3*−/− mice but already 4 weeks of RAS/SGLT2 blockade significantly attenuated this GFR decline (Figure 1C, Supplemental Table 3). Add-on therapy with 1,3-butanediol significantly improved GFR over dual RAS/SGLT2 blockade (Figure 1C), with a mean delta GFR of 80.73 *μ*l/min between these groups. Consistently, BUN levels were lower in the triple versus the dual therapy group (Figure 1D), whereas the urine-albumin-to- creatinine ratio did not show an additive effect (Figure 1E). There was a trend toward a slower body weight loss in *Col4a3*−/− mice on triple therapy, which translated into a longer lifespan of some mice, but mean survival due to uremia did not reach a statistically significant difference versus *Col4a3*−/− mice on dual therapy (Figure 1, F and G). These effects did not differ significantly by sex in stratified Kaplan-Meier analyses (*P* > 0.05; Supplemental Figure 1). However, when comparing GFR, weight curves, and lifespan between male and female mice of the intervention groups, adding BHB seems to have a greater effect on these parameters of female mice on dual blockade (Supplemental Figure 2). There was no additional BHB effect on serum potassium or phosphate levels (Supplemental Figure 3).

**Table 1:** Baseline characteristics at the start of treatment in *Col4a3*−/− mice for lifespan and GFR analysis

Group	Vehicle	RASi+SGLT2i	RASi+SGLT2i+1,3-Butanediol	*P* Value
*n*	20	20	16	
Male, %	50	50	50	
Weight, g	19.8±1.7	20.4±2.5	19.7±3.1	0.6929
GFR, *μ*l/min	181±32	185±38	202±37	0.1744

RASi, renin-angiotensin system inhibitors; SGLT2i, sodium-glucose transporter 2 inhibitors.

**Figure 2:**
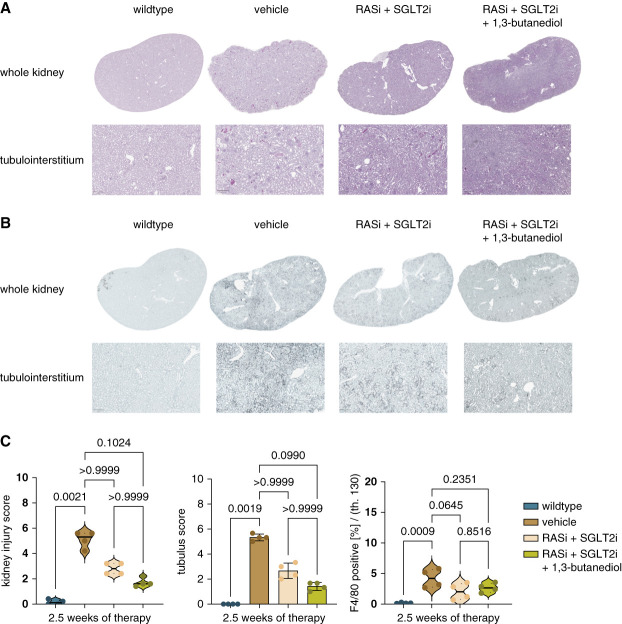
**Tissue morphology and different scores from PAS staining and total area of F4/80 in whole kidney slides in** ***Col4a3**−/−* **mice in wildtype, vehicle, RASi+SGLT2i, and RASi+SGLT2i+1,3-butanediol groups.** (A) Representative PAS-stained images from the whole kidney and tubulointerstitium. (B) Representative F4/80-stained images from the whole kidney and tubulointerstitium. (C) Tubulus and kidney injury scores in PAS staining and F4/80 positive area in whole kidney slides. Testing for significance was performed through ANOVA and Turkey multiple comparison test. Reference bar within each picture represents a length of 200 *μ*m. All quantitative data are means±SD. *P* value of <0.05 was considered to indicate statistical significance. PAS, periodic acid–Schiff.

**Figure 3:**
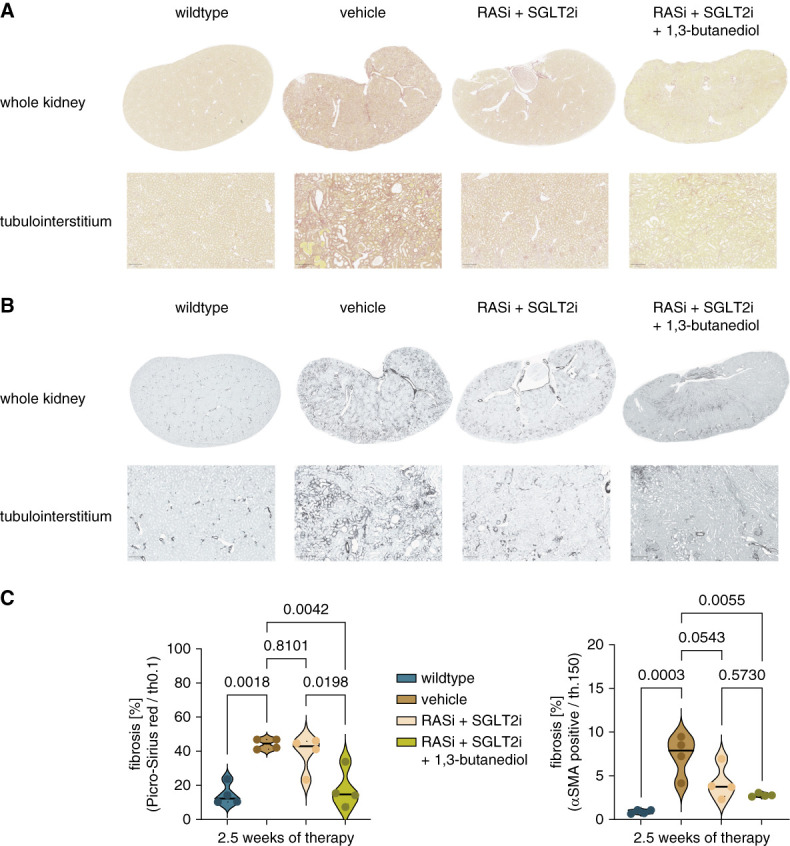
**Tissue morphology and total area of fibrosis in Picro-Sirius red and *α*SMA stained whole kidney slides in** ***Col4a3**−/−* **mice in wildtype, vehicle, RASi+SGLT2i, and RASi+SGLT2i+1,3-butanediol groups.** (A and B) Representative Picro-Sirius red and *α*SMA stained images from the whole kidney and tubulointerstitium. (C) Total area of fibrosis was calculated in whole kidney slides in Picro-Sirius red and *α*SMA staining. Testing for significance was performed through ANOVA and Turkey multiple comparison test. Reference bar within each picture represents a length of 200 *μ*m. All quantitative data are means±SD. *P* value of <0.05 was considered to indicate statistical significance. *α*SMA, alpha smooth muscle actin.

Next, we assessed macroscopic and microscopic kidney morphology in small subsets of mice after 2.5 weeks of each treatment. Macroscopic morphology and kidney weight did not differ between triple and dual therapy groups (Supplemental Figure 4). In addition, most microscopic morphology assessment, such as PAS staining and F4/80 staining, did not differ between the treatment groups (Figure 2). However, triple therapy was more potent than dual therapy in preventing Picro-Sirius red and *α*SMA-positive interstitial fibrosis (Figure 3). In p57 staining, no significant reduction in podocyte loss was detected, but with dual therapy, podocyte numbers were already close to those of wild-type mice (Supplemental Figure 5).

**Figure 4:**
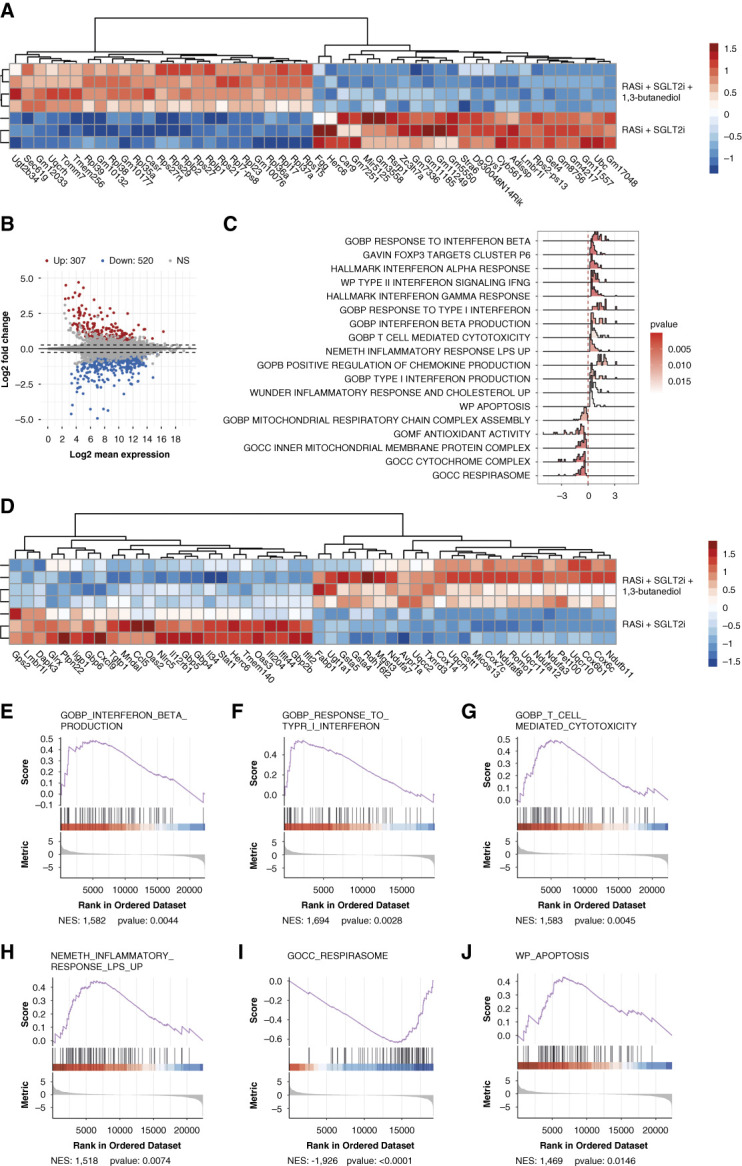
**Bulk RNA-seq of whole kidneys of Alport mice with RASi/SGLT2i or RASi/SGLT2i/1,3-butanediol therapy.** (A) Heatmap presents the biologic replicates of the top genes with differing expression levels between the Alport mice on RASi+SGLT2i and RASi+SGLT2i+1,3-butanediol treatment. Heatmap and dendrogram were used to display the differentially expressed genes' z-scores of normalized counts. (B) A mean-average (MA) plot was also created to display the shrink log2 fold change between kidneys of RASi+SGLT2i treatment versus RASi+SGLT2i+1,3-butanediol treatment. Genes differentially regulated in a significant manner, as indicated by an adjusted *P* value, are labeled in either red or blue. (C) Density ridge plot shows the gene expression distribution of core-enriched genes in enriched gene sets, with gradient color indicating adjusted *P* values using the Benjamini-Hochberg method. (D) Heatmap presents the top differentially expressed genes of the GSEA analysis results between the Alport mice on RASi+SGLT2i and RASi+SGLT2i+1,3-butanediol treatment. Heatmap and dendrogram were used to display the differentially expressed genes' z-scores of normalized counts. (E–J) Selected enrichment plots from the GSEA analysis on the basis of the gene enrichment profiles of the RASi+SGLT2i versus RASi+SGLT2i+1,3-butanediol therapy, NES are displayed. Plots highlight the enrichment for transcriptional signatures related to inflammation, apoptosis, and mitochondrial changes. GAVIN, Gene Annotation and Variant Interpretation; GOCC, Gene Ontology Cellular Component; GOBP, Gene Ontology Biological Process; GOMF, Gene Ontology Molecular Function; GSEA, Gene Set Enrichment Analysis; NES, normalized enrichment scores; UP, upregulated; WP, WikiPathways.

**Figure 5:**
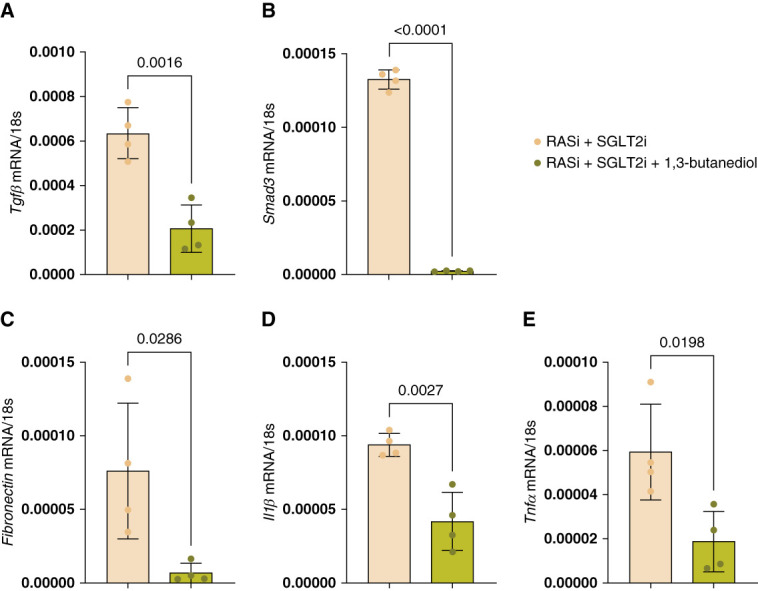
**Real-time qPCR validation of differentially regulated genes.** Gene expression was analyzed by RT-qPCR for fibrosis makers (A) *Tgfβ*, (B) *Smad3*, (C) *Fibronectin*, inflammation marker, (D) *Il1β* and kidney injury marker, and (E) *Tnfα*. Testing for significance was performed by either an unpaired *t* test for normally distributed data or a Mann–Whitney test for non-normally distributed data. All quantitative data are means±SD. *P* value of <0.05 was considered to indicate statistical significance. qPCR, quantitative PCR.

To gain further insights into the additive protective effect of BHB, we performed transcriptome analysis on kidneys obtained after 2.5 weeks of therapy. Cluster analysis clearly separated triple from dual therapy (Figure 4, A and B). RNA sequencing analysis indicated strong effects of BHB on residual inflammation and immune cell response in *Col4a3*−/− mice on dual RAS/SGLT2 blockade (Figure 4, C–J). Furthermore, RNA sequencing indicated positive effects on mitochondrial genes. However, separate RNA sequencing analysis of all mitochondrial genes did not clearly separate between treatment groups (Supplemental Figure 6). NLRP3 expression was not significantly altered by triple therapy (Supplemental Figure 7). Quantitative PCR confirmed the bulk RNA sequencing data, indicating that the addition of BHB to dual therapy further reduced residual tissue fibrosis, inflammation, and kindey injury in *Col4α3 -/-* mice by downregulating the expression of *Tgfβ*,* Smad3*, *Fn*, *Il1β* and *Tnfα.* (Figure 5 and Supplemental Figure 8).

## Discussion

We had hypothesized that oral BHB supplementation added to dual RAS/SGLT2 inhibition would attenuate the progressive loss of GFR. Indeed, our data show that ketone supplementation to mice with CKD on dual RAS/SGLT2 inhibition modulates residual kidney inflammation and fibrosis and sustains GFR. However, these renoprotective effects had only a minor effect on the overall lifespan of *Col4a3*−/− mice with Alport nephropathy, probably because other factors determine the well-being criteria for termination, which were not affected by BHB. Animal welfare required termination of mice at the earliest signs of symptomatic disease, which may not necessarily always relate to kidney structure or GFR. In any case, the effect size of BHB supplementation does not compare with adding the mineralocorticoid receptor antagonists finerenone to dual RAS/SGLT2 blockade in the same model, which was much more potent in prolonging the lifespan of *Col4a3*−/− mice.^25^ Nevertheless, our study is the first to document the effect size of BHB supplementation beyond dual RAS/SGLT2 blockade consistent with data raised with BHB as monotherapy in other mouse models of CKD.^8–10^ For example, we previously demonstrated that BHB attenuated the linear decline of GFR in a mouse model of oxalate nephropathy.^11^ This effect was related to the capacity of BHB to suppress the activation of the NLRP3 inflammasome in intrarenal myeloid cells, namely, tissue macrophages, although this effect was found to be independent from the NLRP3-induced cytokine IL-1*β*.^11^ The capacity of BHB to modulate the phenotype of proinflammatory tissue macrophages and fibroblast activation and proliferation seemed to apply also to the mouse model of Alport nephropathy studied here.^11^ Although we did not perform detailed mechanistic studies, the kidney transcriptome analysis and RT-PCR documents that BHB suppresses residual kidney inflammation and fibrosis even beyond dual RAS/SGLT2 blockade. When the body enters nutritional ketosis, which can be induced by the metabolism of compounds such as 1,3-butanediol, BHB becomes the primary circulating ketone body. BHB is not just an alternative energy source for tissues, but also has potent anti-inflammatory effects. BHB has been shown to inhibit the NLRP3 inflammasome, a key regulator of inflammation, which is known to be involved in kidney injury and fibrosis.^11^ We did not find much change in NLRP3 expression with 1,3-butanediol supplementation, but this does not preclude effects on its enzymatic activation. In addition, we have previously described that BHB can modulate fibroblast activation, a key event in the progression of kidney fibrosis, by altering the TGF-*β* signaling pathway.^11^ This may result in reduced collagen deposition and improved tissue architecture in the kidney. The reduced fibrotic burden can directly correlate with better kidney function, as it leads to less scarring and better preservation of glomerular and tubulointerstitial structures. In addition, the metabolic shift toward ketosis seemed to affect mitochondrial function. Our transcriptome analysis found a profound down regulation of ribosomal protein genes regulating protein synthesis, which suggests that 1,3-butanediol treatment influences residual cellular activity and response to injury beyond standard-of-care therapy. We also found a profound regulation of genes within still uncharacterized regions of the genome, suggesting their involvement in regulatory networks influenced by the intervention. By impacting on these mechanisms, BHB seems to alleviate the detrimental effects on kidney function caused by excessive inflammation, thereby supporting improved GFR. This reduction of inflammation and fibrosis is likely to be the reason for a retarded loss of GFR when adding BHB. However, the observed effects of BHB did not translate into a significant prolongation of the mean (kidney) lifespan of *Col4a3*−/− mice.

Our study has several limitations. According to the available permissions, we could obtain only a limited number of animals for the mechanistic studies after 2.5 weeks of therapy. The small number may not fully capture what happened in most animals. Additional time points could have addressed the possibility that the effects of BHB could only be transient and vane after 2–3 weeks, but such a permission could not be obtained. In addition, the experiment included mice of both sexes to portray human CKD populations. Surprisingly, females seem to benefit more from BHB supplementation than male mice. This subgroup analysis must be interpreted with caution because the study was not designed and lacked power to analyze this aspect. Currently, nothing is known about sex-specific effects of ketone supplements, but this finding may encourage further investigations relating this topic. Higher doses of BHB may have been more potent, but are also less well tolerated, and the present BHB exposure resulted in significant ketosis in all animals studied. The latter also excludes that the animals reduced food intake for changes in taste of the food, but also weights were comparable among the groups and only declined in the phase of uremia. However, the most relevant limitation of our study is that we only assessed effects of late initiation of dual or triple therapy, and effect size of BHB supplementation may have been much larger if treatment would have been initiated earlier, as demonstrated for RAS inhibition in this model.^26^ Thus, the results reported here leave uncertainties regarding the possible efficacy and toxicity of BHB supplementation as part of a triple therapy in humans, which remains to be tested in a human randomized controlled trial. Likely, effect size will be different in other forms of nondiabetic kidney disease.

Together, ketone supplementation significantly attenuated the decline of GFR in *Col4a3−/−* mice beyond dual RAS/SGLT2 inhibition. However, the effect on (kidney) survival was limited. Earlier onset of treatment might increase the reported effects but, even then, is not likely to compare with the effects of triple therapy including finerenone reported earlier. Therefore, we would prioritize clinical trials with more potent combinations therapies than RASi/SGLT2i and BHB.

## Supplementary Material

## Data Availability

Data related to transcriptomic, proteomic, or metabolomic data; All data are included in the manuscript and/or supporting information; Previously published data were used for this study. Raw Data/Source Data. BioProject Accession; Gene Expression Omnibus. GEO: GSE226353 and GSE287708 (reviewer access: wduvcccyvzizruj). PRJNA940042. The RNAseq data supporting the findings of this study are openly available on publication. The RNAseq data are available in the Gene Expression Omnibus database (GSE226353) and (GSE287708). The raw data are available in the Sequence Read Archive associated with the projects. All data are included in the manuscript or the provided supplement.

## Supplemental Material

Supplemental File 1. Description of experimental animals and study design.

Supplemental File 2. Animal welfare surveillance.

Supplemental File 3. Immunostaining with p57 and deep learning analysis.

Supplemental File 4. Primers used for real-time qRT-PCR.

Supplemental Table 1. Baseline characteristics at the start of treatment in male *Col4a3*−/− mice for lifespan and GFR analysis.

Supplemental Table 2. Baseline characteristics at the start of treatment in female *Col4a3*−/− mice for lifespan and GFR analysis.

Supplemental Table 3. Evaluation of GFR in *Col4a3*−/− mice.

Supplemental Figure 1. Sex-specific Kaplan-Meier plots.

Supplemental Figure 2. Sex-specific analysis of GFR, lifespan, and weight loss between intervention groups.

Supplemental Figure 3. Effect of different treatments on potassium and phosphate.

Supplemental Figure 4. Macroscopic kidney atrophy.

Supplemental Figure 5. p57 staining.

Supplemental Figure 6. RNAseq for mitochondrial genes.

Supplemental Figure 7. NLRP3 gene expression.

Supplemental Figure 8. qPCR.
