# Supplementary material for: Oral Ketone β-Hydroxybutyrate Supplement Retards the Loss of GFR in Alport Mice on Dual Renin-Angiotensin System/Sodium-Glucose Transporter 2 Blockade
Source: Kidney360. 2025 Mar 11;6(7):1085–95. doi: 10.34067/KID.0000000747 (PMC12338352; doi:10.34067/KID.0000000747)
Supplement: Supplementary file 1 [file kidney360-6-1085-s001.pdf]

ASN American Society of Nephrology
JASN Journal of the American Society of Nephrology
KIDNEY360 Accessing Our World From Every Angle
CJASN Clinical Journal of the American Society of Nephrology

ASN Journal Disclosure Form

As per ASN journal policy, I have disclosed any financial relationships or commitments I have held in the past 36 months as included below. I have listed my Current Employer below to indicate there is a relationship requiring disclosure. If no relationship exists, my Current Employer is not listed.

H. Anders reports the following:
Employer: Klinikum der Universitat Munchen; Consultancy: AstraZeneca, Novartis, GSK, Bayer, Roche, Otsuka, Vifor, Boehringer-Ingelheim, SOBI; Research Funding: Boehringer-Ingelheim; and Advisory or Leadership Role: Editor-in-Chief NDT.

I understand that the information above will be published within the journal article, if accepted, and that failure to comply and/or to accurately and completely report the potential financial conflicts of interest could lead to the following: 1) Prior to publication, article rejection, or 2) Post-publication, sanctions ranging from, but not limited to, issuing a correction, reporting the inaccurate information to the authors' institution, banning authors from submitting work to ASN journals for varying lengths of time, and/or retraction of the published work.

Name: Hans J. Anders
Manuscript ID: K360-2024-000896R1
Manuscript Title: Oral ketone beta-hydroxybutyrate supplement retards the loss of glomerular filtration rate in Alport mice on dual RAS/SGLT2 blockade
Date of Completion: January 31, 2025
Disclosure Updated Date: October 18, 2024

ASN American Society of Nephrology JASN Journal of the American Society of Nephrology KIDNEY360 Accessing Our World From Every Angle CJASN Clinical Journal of the American Society of Nephrology

ASN Journal Disclosure Form

As per ASN journal policy, I have disclosed any financial relationships or commitments I have held in the past 36 months as included below. I have listed my Current Employer below to indicate there is a relationship requiring disclosure. If no relationship exists, my Current Employer is not listed.

M. Klaus reports the following:
Employer: University Hospital LMU Munich

I understand that the information above will be published within the journal article, if accepted, and that failure to comply and/or to accurately and completely report the potential financial conflicts of interest could lead to the following: 1) Prior to publication, article rejection, or 2) Post-publication, sanctions ranging from, but not limited to, issuing a correction, reporting the inaccurate information to the authors' institution, banning authors from submitting work to ASN journals for varying lengths of time, and/or retraction of the published work.

Name: Martin Klaus
Manuscript ID: K360-2024-000896R1
Manuscript Title: Oral ketone beta-hydroxybutyrate supplement retards the loss of glomerular filtration rate in Alport mice on dual RAS/SGLT2 blockade
Date of Completion: January 27, 2025
Disclosure Updated Date: April 29, 2024

ASN
American Society of Nephrology
JASN
Journal of the American Society of Nephrology
KIDNEY360
Accessing Our World From Every Angle
CJASN
Clinical Journal of the American Society of Nephrology

ASN Journal Disclosure Form

As per ASN journal policy, I have disclosed any financial relationships or commitments I have held in the past 36 months as included below. I have listed my Current Employer below to indicate there is a relationship requiring disclosure. If no relationship exists, my Current Employer is not listed.

J. Ku reports the following:
Employer: Daglfinger Blumenhof, LingoAce, LMU Klinikum; and Ownership Interest: Tesla, Nvidia, ETFs, etc.

I understand that the information above will be published within the journal article, if accepted, and that failure to comply and/or to accurately and completely report the potential financial conflicts of interest could lead to the following: 1) Prior to publication, article rejection, or 2) Post-publication, sanctions ranging from, but not limited to, issuing a correction, reporting the inaccurate information to the authors' institution, banning authors from submitting work to ASN journals for varying lengths of time, and/or retraction of the published work.

Name: John Ku
Manuscript ID: K360-2024-000896R1
Manuscript Title: Oral ketone beta-hydroxybutyrate supplement retards the loss of glomerular filtration rate in Alport mice on dual RAS/SGLT2 blockade
Date of Completion: January 24, 2025
Disclosure Updated Date: January 24, 2025

ASN American Society of Nephrology | JASN Journal of the American Society of Nephrology | KIDNEY360 Accessing Our World From Every Angle | CJASN Clinical Journal of the American Society of Nephrology

ASN Journal Disclosure Form

As per ASN journal policy, I have disclosed any financial relationships or commitments I have held in the past 36 months as included below. I have listed my Current Employer below to indicate there is a relationship requiring disclosure. If no relationship exists, my Current Employer is not listed.

Y. Kusunoki reports the following:
Employer: Hokkaido university hospital

I understand that the information above will be published within the journal article, if accepted, and that failure to comply and/or to accurately and completely report the potential financial conflicts of interest could lead to the following: 1) Prior to publication, article rejection, or 2) Post-publication, sanctions ranging from, but not limited to, issuing a correction, reporting the inaccurate information to the authors' institution, banning authors from submitting work to ASN journals for varying lengths of time, and/or retraction of the published work.

Name: Yoshihiro Kusunoki
Manuscript ID: K360-2024-000896R1
Manuscript Title: Oral ketone beta-hydroxybutyrate supplement retards the loss of glomerular filtration rate in Alport mice on dual RAS/SGLT2 blockade.
Date of Completion: January 24, 2025
Disclosure Updated Date: January 24, 2025

ASN American Society of Nephrology
JASN Journal of the American Society of Nephrology
KIDNEY360 Accessing Our World From Every Angle
CJASN Clinical Journal of the American Society of Nephrology

ASN Journal Disclosure Form

As per ASN journal policy, I have disclosed any financial relationships or commitments I have held in the past 36 months as included below. I have listed my Current Employer below to indicate there is a relationship requiring disclosure. If no relationship exists, my Current Employer is not listed.

C. Li reports the following:
Employer: University of Pennsylvania

I understand that the information above will be published within the journal article, if accepted, and that failure to comply and/or to accurately and completely report the potential financial conflicts of interest could lead to the following: 1) Prior to publication, article rejection, or 2) Post-publication, sanctions ranging from, but not limited to, issuing a correction, reporting the inaccurate information to the authors' institution, banning authors from submitting work to ASN journals for varying lengths of time, and/or retraction of the published work.

Name: Chenyu Li
Manuscript ID: K360-2024-000896R1
Manuscript Title: Oral ketone beta-hydroxybutyrate supplement retards the loss of glomerular filtration rate in Alport mice on dual RAS/SGLT2 blockade,
Date of Completion: January 24, 2025
Disclosure Updated Date: January 24, 2025

ASN American Society of Nephrology
JASN Journal of the American Society of Nephrology
KIDNEY360 Accessing Our World From Every Angle
CJASN Clinical Journal of the American Society of Nephrology

ASN Journal Disclosure Form

As per ASN journal policy, I have disclosed any financial relationships or commitments I have held in the past 36 months as included below. I have listed my Current Employer below to indicate there is a relationship requiring disclosure. If no relationship exists, my Current Employer is not listed.

L. Schreier reports the following:
Employer: Medizinische Klinik und Poliklinik IV der Ludwig Maximilians Universität Abteilung Nephrologie

I understand that the information above will be published within the journal article, if accepted, and that failure to comply and/or to accurately and completely report the potential financial conflicts of interest could lead to the following: 1) Prior to publication, article rejection, or 2) Post-publication, sanctions ranging from, but not limited to, issuing a correction, reporting the inaccurate information to the authors' institution, banning authors from submitting work to ASN journals for varying lengths of time, and/or retraction of the published work.

Name: Linus Paul Schreier
Manuscript ID: K360-2024-000896R1
Manuscript Title: Oral ketone beta-hydroxybutyrate supplement retards the loss of glomerular filtration rate in Alport mice on dual RAS/SGLT2 blockade.
Date of Completion: January 24, 2025
Disclosure Updated Date: January 24, 2025

ASN American Society of Nephrology
JASN Journal of the American Society of Nephrology
KIDNEY360 Accessing Our World From Every Angle
CJASN Clinical Journal of the American Society of Nephrology

ASN Journal Disclosure Form

As per ASN journal policy, I have disclosed any financial relationships or commitments I have held in the past 36 months as included below. I have listed my Current Employer below to indicate there is a relationship requiring disclosure. If no relationship exists, my Current Employer is not listed.

Z. Zhu reports the following:
Employer: Beijing Anzhen Hospital, Capital Medical University

I understand that the information above will be published within the journal article, if accepted, and that failure to comply and/or to accurately and completely report the potential financial conflicts of interest could lead to the following: 1) Prior to publication, article rejection, or 2) Post-publication, sanctions ranging from, but not limited to, issuing a correction, reporting the inaccurate information to the authors' institution, banning authors from submitting work to ASN journals for varying lengths of time, and/or retraction of the published work.

Name: Zhihui Zhu
Manuscript ID: K360-2024-000896R1
Manuscript Title: Oral ketone beta-hydroxybutyrate supplement retards the loss of glomerular filtration rate in Alport mice on dual RAS/SGLT2 blockade
Date of Completion: February 27, 2025
Disclosure Updated Date: February 27, 2025
