# Supplementary material for: Oral Ketone β-Hydroxybutyrate Supplement Retards the Loss of GFR in Alport Mice on Dual Renin-Angiotensin System/Sodium-Glucose Transporter 2 Blockade
Source: Kidney360. 2025 Mar 11;6(7):1085–95. doi: 10.34067/KID.0000000747 (PMC12338352; doi:10.34067/KID.0000000747)
Supplement: Supplementary file 2 [file kidney360-6-1085-s002.pdf]

SUPPLEMENTARY APPENDIX

for

Oral ketone beta-hydroxybutyrate supplement retards the loss of glomerular filtration rate in Alport mice on dual RAS/SGLT2 blockade

by

Linus P. Schreier, Zhihui Zhu, Yoshihiro Kusunoki, Chenyu Li, John Ku, Martin Klaus, Hans-Joachim Anders

contents

Supplementary file 1: Description of experimental animals and study design 3

Supplementary file 2: Animal welfare surveillance 6

Supplementary file 3: Immunostaining with p57 and deep learning analysis 7

Supplementary file 4: Primers used for real time qRT-PCR 9

Supplementary table 1: Baseline characteristics at the start of treatment in male *Col4a3-/-* mice for lifespan and GFR analysis 10

Supplementary table 2: Baseline characteristics at the start of treatment in female *Col4a3-/-* mice for lifespan and GFR analysis 10

Supplementary table 3: Evaluation of GFR in *Col4a3-/-* mice 10

Supplementary figure 1: Sex specific Kaplan Meier plots 11

Supplementary figure 2: Sex specific analysis of GFR, lifespan and weight loss between intervention groups 12

Supplementary figure 3: Effect of different treatments on potassium and phosphate 13

Supplementary figure 4: Macroscopic kidney atrophy 14

Supplementary figure 5: p57 staining 15

Supplementary figure 6: RNAseq for mitochondrial genes 16

Supplementary figure 7: NLRP3 gene expression 16

Supplementary figure 8: qPCR 17

Supplementary file 1: Description of experimental animals and study design

Experimental animals

Col4a3-/- (Col4a3tm1Dec) mice with a 129/SvJ background (Jackson Lab, Bar Harbor, ME, USA) are a widely used animal model of progressive CKD in Alport syndrome. In this study 50% female and 50% male mice were used. We included 6-week-old mice in the study, these mice already suffered from a late stage of kidney disease. These mice were immune competent and did not undergo any prior procedures before being included in the experiment.

Inclusion criteria and exclusion criteria

Inclusion criteria were as follows: Male and female mice, six weeks of age. The exclusion criteria were the following: a score ≥ 2 for more than 6h during animal welfare surveillance (supplementary file 2); exposure to any other drug during the study period, e.g. treatment requested by the veterinarian for skin wounds; any sign of infection; pregnancy; signs of drug intolerance (anaphylactic reactions). As only Col4a3-/- mice were included in the analysis genotyping was done before and after the experiment with two different samples of the same mouse to ensure the correct genotype. In the RASi + SGLT2i + 1,3-butanediol group one heterozygous mouse was falsely include but later excluded when the genotyping was done for the second time.

Housing and husbandry

129-Col4a3tm1Dec-/- mice with spontaneous CKD (Alport nephropathy) were used in this study and were bred in an in-house colony. All mice were kept under pathogen-free conditions in a 12-hour light and dark cycle with free access to food and water. Enrichment was provided as per local regulatory requirements. Health status, cleaning, beddings, water, and food supply were according to our center´s local standard operating procedures.

Study design and schedule

The goal of this placebo-controlled experiment was to evaluate the effect of the BHB precursor 1,3-

butanediol on top of ramipril and empagliflozin on CKD progression in mice with Alport nephropathy. At the age of four weeks the mice were genotyped to select the mice with homozygote Alport Syndrome, as they were bread from heterozygous parents. After an initial GFR measurement at the age of six weeks the mice were fed the drugs “ad libitum” via the food for eight weeks. Mice received either a) vehicle or b) 50 mg/kg ramipril plus 150 mg/kg empagliflozin or c) 50 mg/kg ramipril plus 150 mg/kg empagliflozin plus 200g/kg 1,3-butanediol. During the treatment GFR was measured at the age of six weeks, seven weeks and at the age of ten weeks, also urine was taken weekly. After 2.5 weeks of treatment a subgroup of animals in every group was euthanized for organ and blood taking. In the subgroup analysis 4 mice of every treatment group were compared to each other. During the experiment mice were scored daily to assure animal welfare and to euthanize the mice shortly before they suffered from uraemia. After reaching predefined termination criteria the mice were euthanized, and organs and blood were taken (supplementary file 2). The timepoint of termination was defined as death by uraemia. This study protocol was established in advance but not registered.

Primary and secondary endpoints

The primary endpoint of this study was the overall survival. Animal welfare scoring was performed daily. Upon reaching a predefined score (supplementary file 2), animals were sacrificed and the timepoint was recorded as the day of uremic death. This predefined endpoint reduced the suffering from uraemia in the mice. Tissues and bio samples were harvested to validate end-stage kidney disease as the cause of demise. The secondary endpoints were the markers of kidney excretory function (GFR, proteinuria, BUN). Also, histomorphology and gene expression of the kidney in a subgroup of animals was analysed.

Study schematic

The study schematic is shown below:

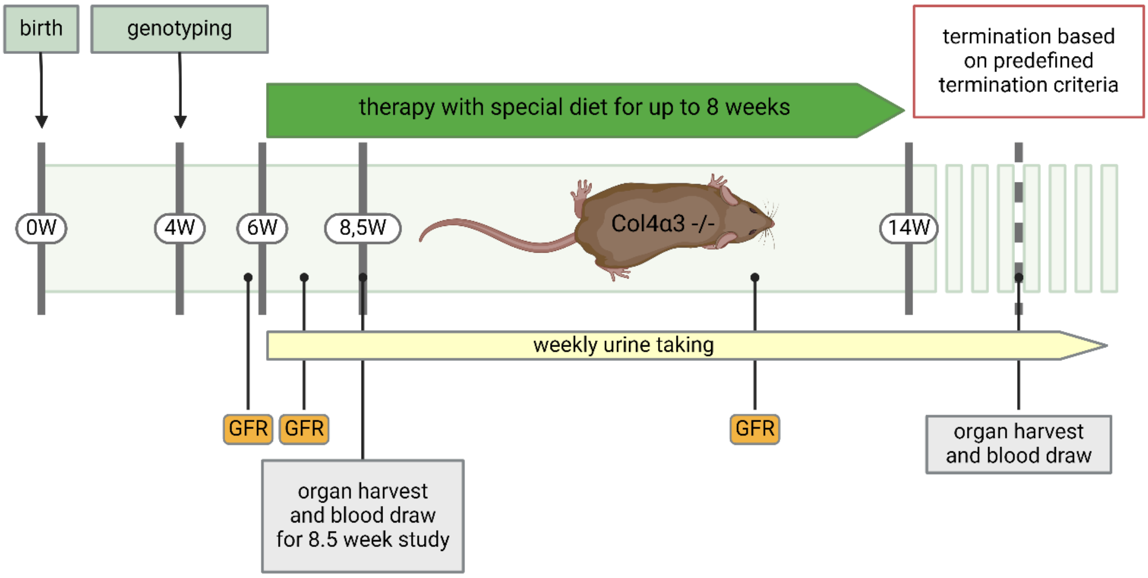

This schematic represents the design and schedule of the conducted experiment, GFR (glomerular filtration rate). This schematic was created with *biorender*.

Supplementary file 2: Animal welfare surveillance

Animal welfare surveillance

**No alterations**	**Mildly altered (1 point)**	**Moderately altered (2 points)**	**Severely altered (4 points)**
Shiny, clean fur<br>Builds nest<br>Social interactions<br>Reacting to stimulus<br>Normal motions<br>Exploratory behaviour<br>GFR＞150 ul/min	Weight loss <10%<br>Partially piloerection<br>Dampened but reactive<br>Transient bent back<br>Transient tremor<br>Transient abnormal respiration<br>Transient wet wound<br>GFR 50-150ul/min	Weight loss <15%<br>Permanent piloerection<br>Dampened on provocation<br>Intermittent bent back<br>Repeated abnormal breathing<br>Intermittent tremor<br>Transient exhaustion·<br>Redness, swelling at the injection site<br>GFR＜50 μl/min	Weight loss >15%<br>Moderate-severe dehydration<br>Not reactive to stimuli<br>Persistent back bending<br>Persistent abnormal breathing<br>Persistent tremor<br>Convulsions<br>Persistent exhaustion<br>Self-destructive injuries<br>Self-isolation

- “transient”: only one time detected in two subsequent checks.
- “intermittent”: in three subsequent checks, 1. timepoint: yes; 2. timepoint: no; 3. timepoint: yes.
- “repeated”: at two timepoints.
- “persistent”: > two timepoints.
- “moderate dehydration”: piloerection and possible cause of mild weight loss. 2 points. Give volume (s.c. NaCl 0.9 % 0.2 - 0.4 ml at max. volume 10 ml/kg in single injection site).
- Termination (cervical dislocation) once 4 points have been reached in summation or at the one timepoint.
- A sum score of 2 or 3 points: repeat assessment after 6 and 12 h. Worsening should lead to consultation with a veterinarian.

Supplementary file 3: Immunostaining with p57 and deep learning analysis

Scanning

All analysed slides were digitalized using the whole slide scanner Aperio GT 450 with the 40x objective (Leica Biosystems, Wetzlar, Germany).

Deep learning morphometry analysis of p57 stained immunohistochemistry slides

Sections were stained with p57 antibody (1:200, Santa Cruz Biotechnology, USA) by immunohistochemistry method, p57 staining was used for podocyte quantification [1]. Subsequently, the slides were digitalized using a whole slide scanner (Aperio GT 450, Leica Biosystems, Wetzlar, Germany).

To train and validate the algorithm, glomeruli and podocytes were annotated in whole kidney slides using QuPath [2]. Subsequently, pretrained U-Net-based deep learning segmentation algorithms [3,4] were trained to segment kidney glomeruli and podocytes (see figure below). Obtained segmentation were filtered and for the morphometric analysis only cell structures within the detected glomeruli were used. Finally, podocyte counts in each glomerulus were computed.

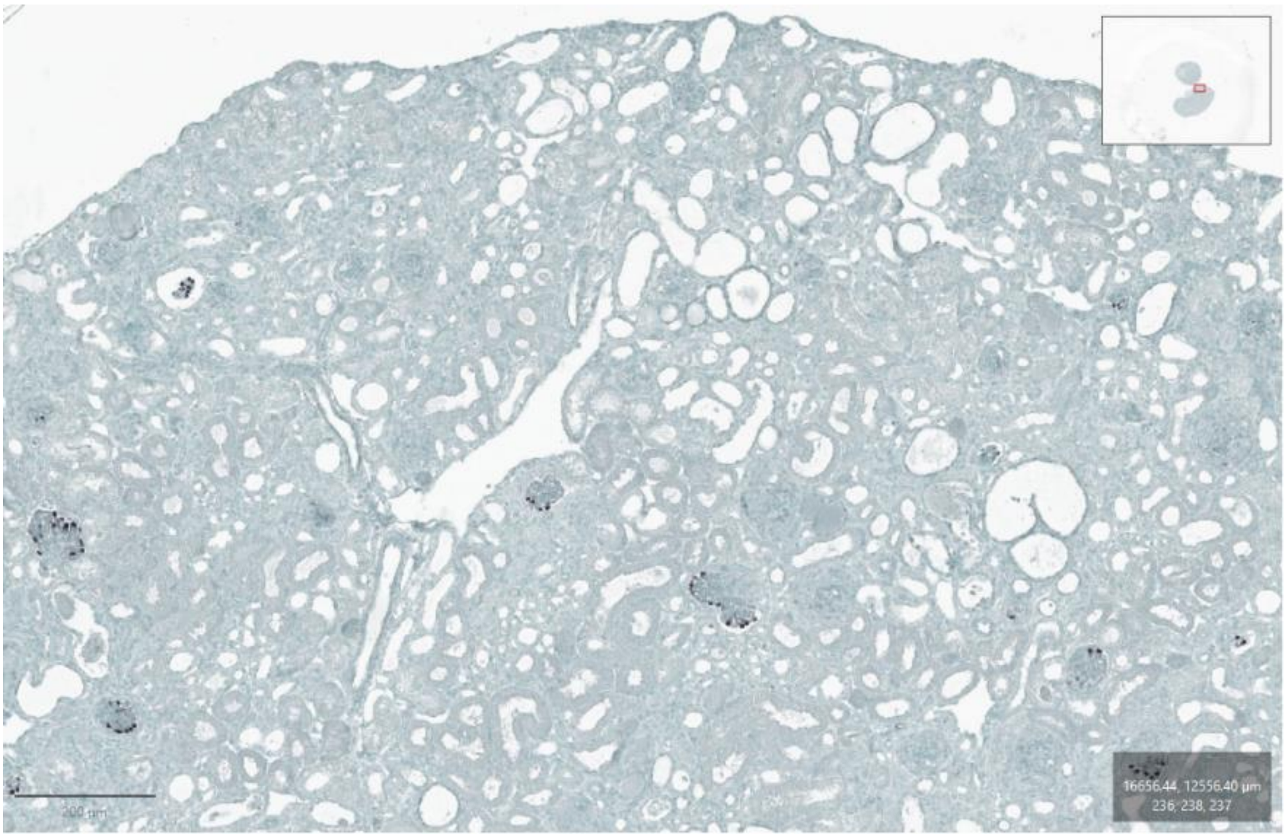

Exemplary section of a p57 stained slide of a CKD-vehicle mouse with severe glomerulosclerosis.

Wildtype **CKD-vehicle**

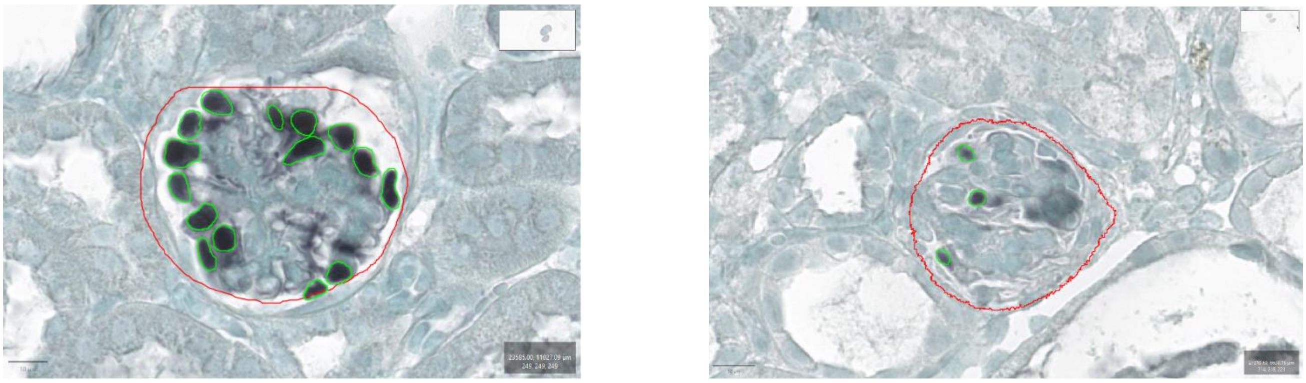

Exemplary section of a p57 stained whole slide image with the detected structures/cells by the deep learning segmentations: Glomerulus (red), podocyte nuclei (green).

Supplementary file 4: Primers used for real time qRT-PCR

Primers used for real time qRT-PCR

Gene	Forward (5'-3')	Reverse (5'-3')
18s	GCAATTATTCCCCATGAACG	AGGGCCTCACTAAACCATCC
NGAL	ATGTCACCTCCATCCTGG	GCCACTTGCACATTGTAG
KIM1	TCAGCTCGGGAATGCACAA	TGGTTGCCTTCCGTGTCTCT
COL1a1	ACATGTTCAGCTTTGTGGAC	TAGGCCATTGTGTATGCAG
Podocin	CTGTGAGTGGCTTCTTGTCCTC	CCTTTGGCTCTTCCAGGAAGCA
Fibronectin	GCCACCATTACTGGTCTGGA	GGTTGGTGATGAAGGGGGTC
TGFβ	CAACCCAGGTCCTTCCTAAA	GGAGAGCCCTGGATACCAAC
TNFα	AGCCTCTTCTCATTCCTGCT	TAGACAAGGTACAACCCATC
Il1b	TGGACCTTCCAGGATGAGGACA	GTTCATCTCGGAGCCTGTAGTG
IL6	TACCACTTCACAAGTCGGAGGC	CTGCAAGTGCATCATCGTTGTTC
CTGF	CACCCGGGTTACCAATGACA	TCCGGGACAGTTGTAATGGC
SMAD3	CTACTGCCACTTGGAGTCTC	AACTGCCCCGTCTTCTTGAG
FOXo3a	GGCAAAGCAGACCCTCAAAC	TGAGAGCAGATTTGGCAAAG

Supplementary table 1: Baseline characteristics at the start of treatment in male *Col4a3-/-* mice for lifespan and GFR analysis

Group	vehicle	RASi + SGLT2i	RASi + SGLT2i + 1,3-butanediol	p
**n**	10	10	8	
**Weight [g]**	21.3 ± 2.7	22.2 ± 1.9	22.3 ± 1.2	0.5002
**GFR [µl/min]**	181 ± 26	199 ± 40	214 ± 46	0.2036

Abbreviations: GFR (glomerular filtration rate)

Supplementary table 2: Baseline characteristics at the start of treatment in female *Col4a3-/-* mice for lifespan and GFR analysis

Group	vehicle	RASi + SGLT2i	RASi + SGLT2i + 1,3-butanediol	p
**n**	10	10	8	
**Weight [g]**	18.7 ± 2.2	18.7 ± 1.7	17.1 ± 1.8	0.1387
**GFR [µl/min]**	180 ± 39	171 ± 32	191 ± 23	0.4534

Abbreviations: GFR (glomerular filtration rate)

Supplementary table 3: Evaluation of GFR in *Col4a3-/-* mice

Group (n)	GFR (µl/min)		
	**Baseline (6 weeks)**	**7 weeks**	**10 weeks**
**Vehicle (20)**	181 ± 32	147 ± 63	0.00 ± 0.00
**RASi + SGLT2i (20)**	185 ± 38	179 ± 31	57 ± 54
**RASi + SGLT2i + 1,3-butanediol (16)**	202 ± 37	192 ± 60	139 ± 69

Abbreviations: GFR (glomerular filtration rate)

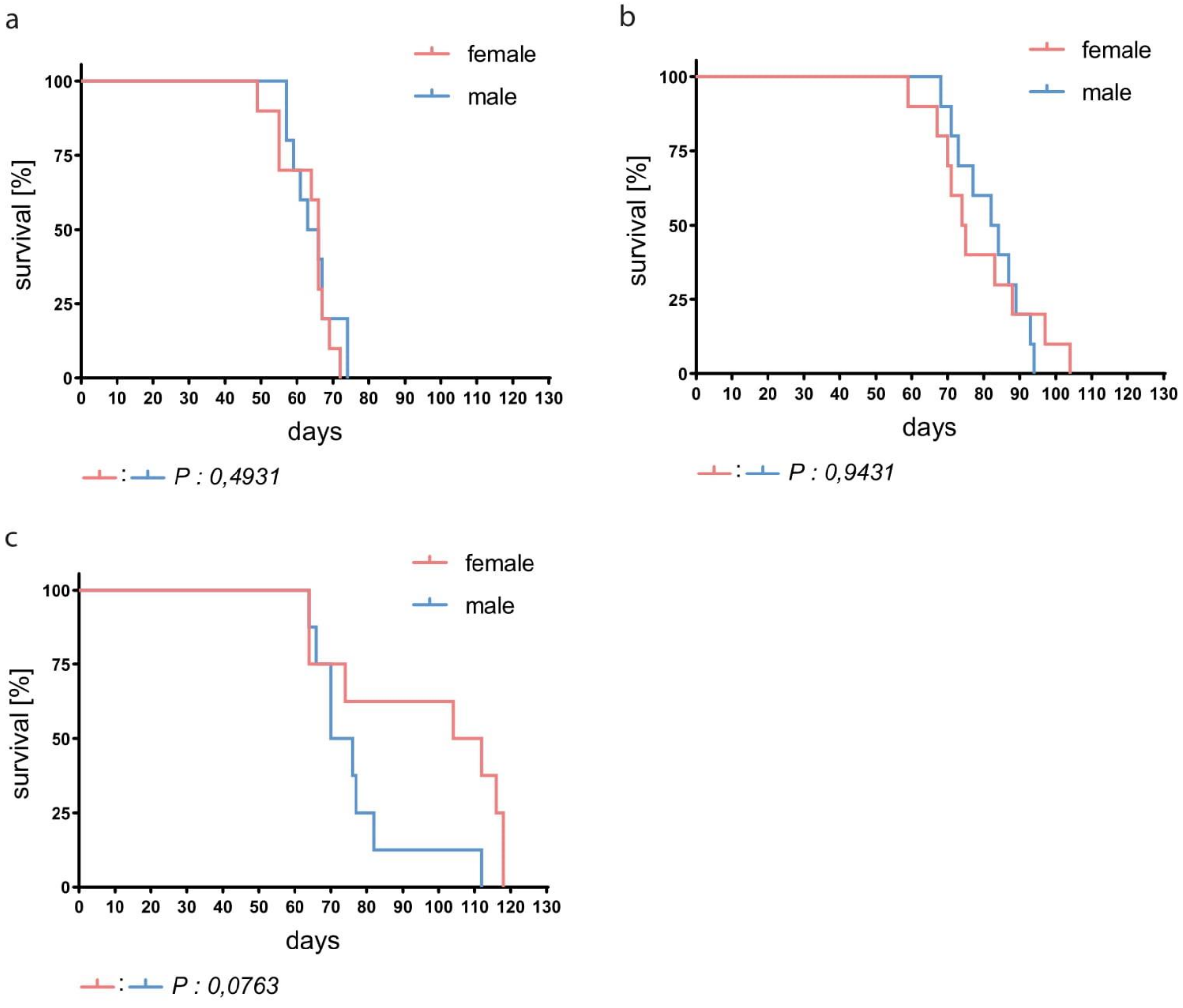

Supplementary figure 1: Sex specific Kaplan Meier plots

(a) Sex differences in vehicle group. (b) Sex differences in RASi + SGLT2i group. (c) Sex differences in RASi + SGLT2i + 1,3-butanediol group. Data is shown in a Kaplan Meier plot, testing for significant differences was carried out via Log-Rank test. A *P* value of <0.05 was considered to indicate statistical significance.

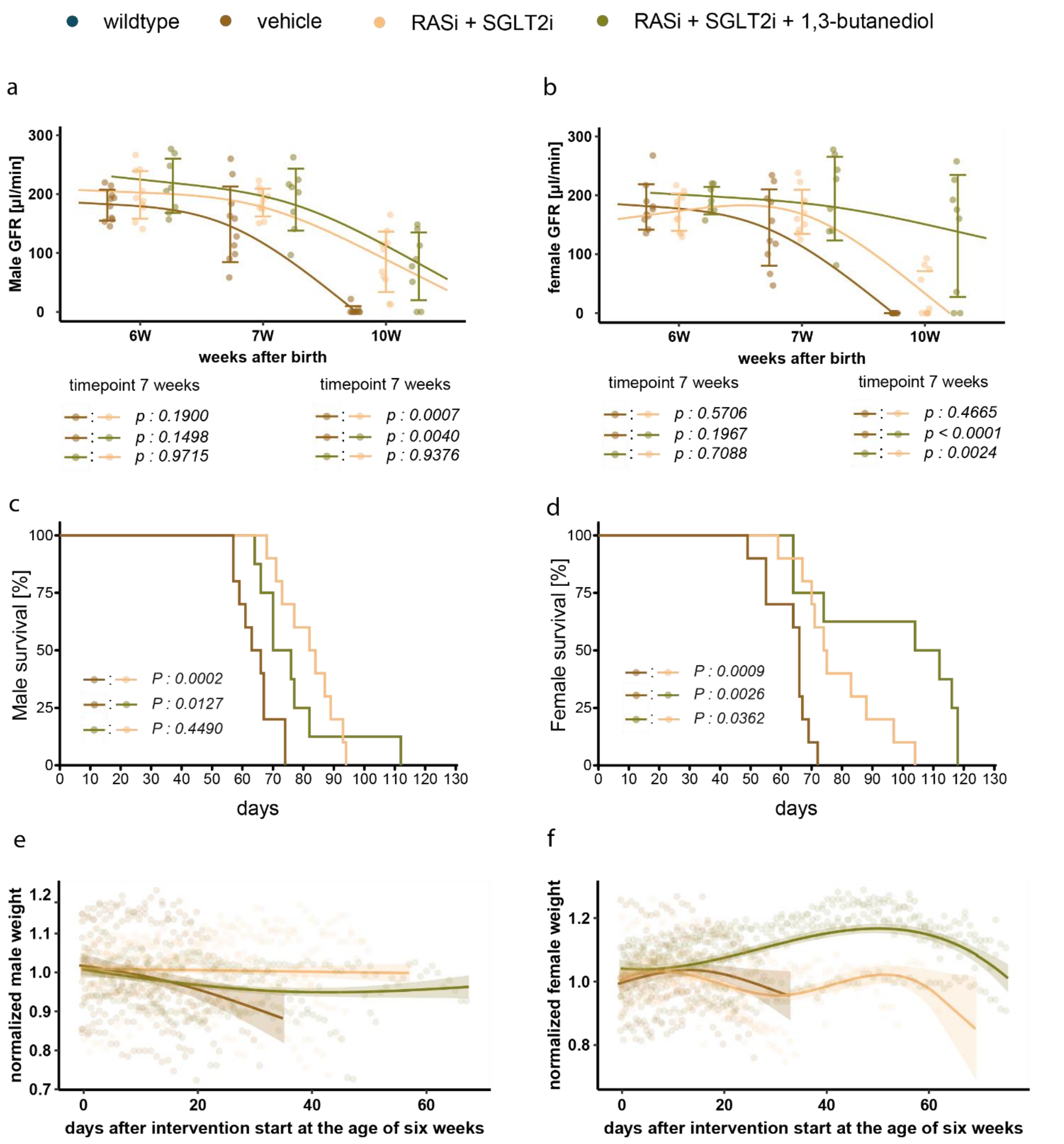

Supplementary figure 2: Sex specific analysis of GFR, lifespan and weight loss between intervention groups

Effects of vehicle, RASi + SGLT2i and RASi + SGLT2i + 1,3-butanediol therapy on GFR decline in (a) males and (b) females. Survival rate (Uraemia-free survival) of vehicle, RASi + SGLT2i and RASi + SGLT2i + 1,3-butanediol group for (c) males and (d) females. Change of bodyweight after therapy start in different groups for (e) males and (f) females. For (a and b) a 2-way ANOVA with following Turkey`s multiple comparison test was used for the timepoints 7 and 10 weeks. For the comparison of the survival curves in (c and d) a Kaplan Meier analysis was used. All quantitative data are means ± SD. A P value of <0.05 was considered to indicate statistical significance.

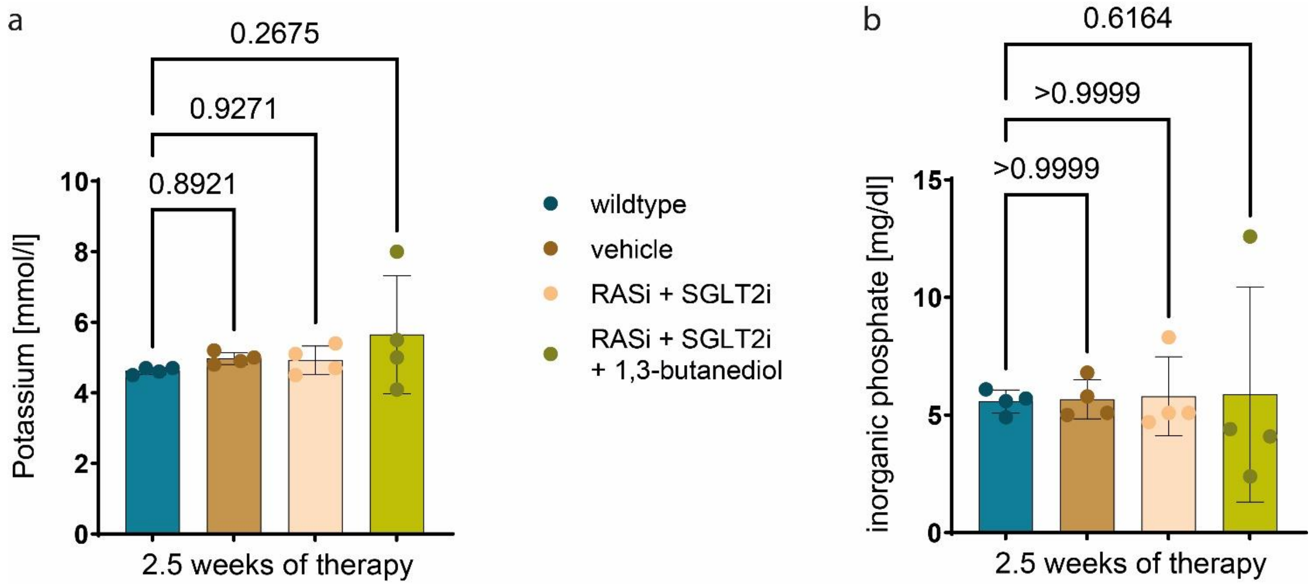

Supplementary figure 3: Effect of different treatments on potassium and phosphate

(a) The serum levels of potassium after 2.5 weeks of therapy. (b) The serum level of inorganic phosphate after 2.5 weeks of therapy. Testing for significance was carried out via ANOVA and Dunn's test for multiple comparisons. All quantitative data are means ± SD. A *P* value of <0.05 was considered to indicate statistical significance.

a

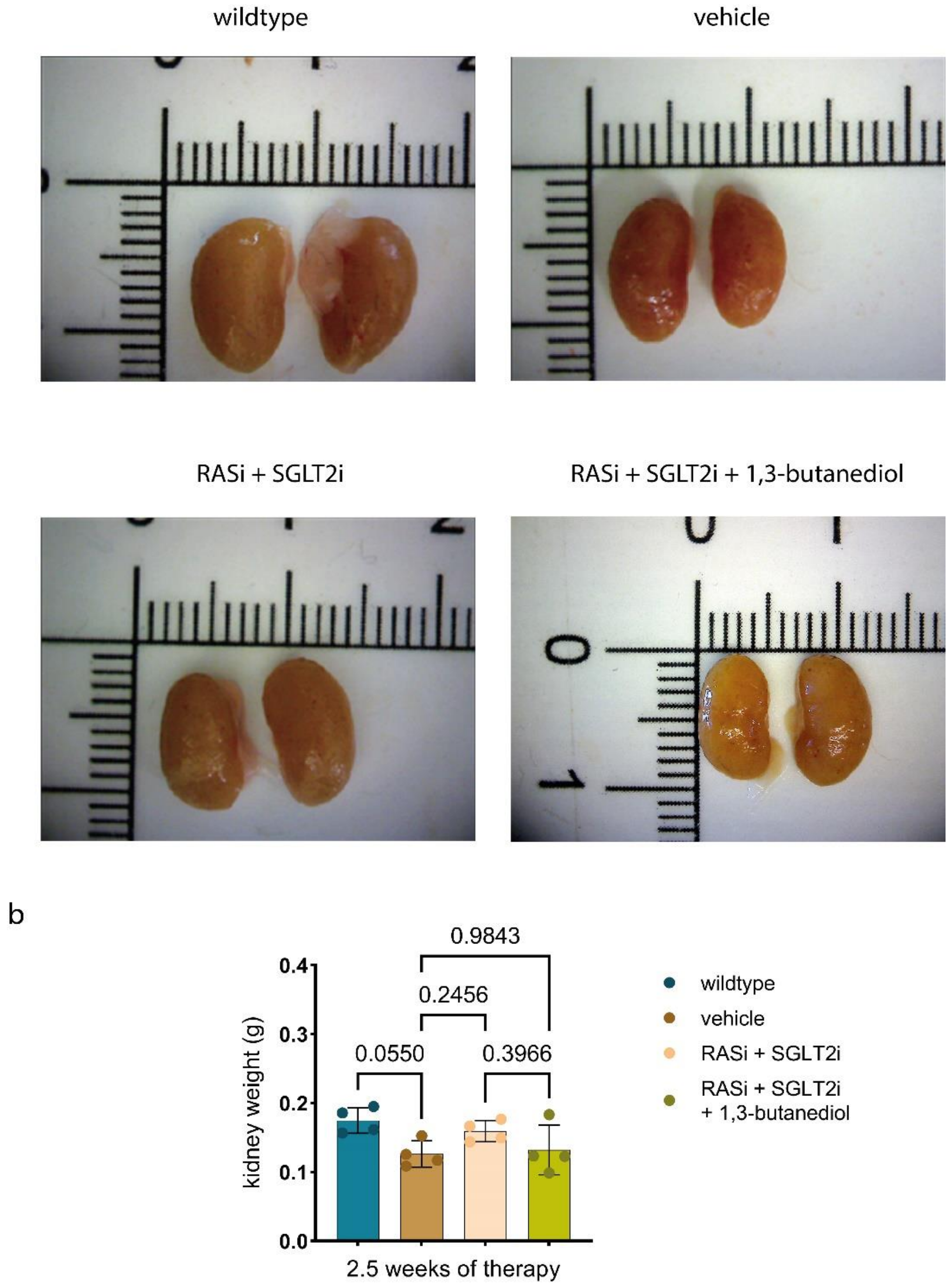

Supplementary figure 4: Macroscopic kidney atrophy

(a) Representative kidney images after 2.5 weeks of therapy. (b) kidney weight after 2.5 weeks of therapy. Testing for significance was carried out via ANOVA and Turkey´s test for multiple comparisons. All quantitative data are means ± SD. A *P* value of <0.05 was considered to indicate statistical significance.

a

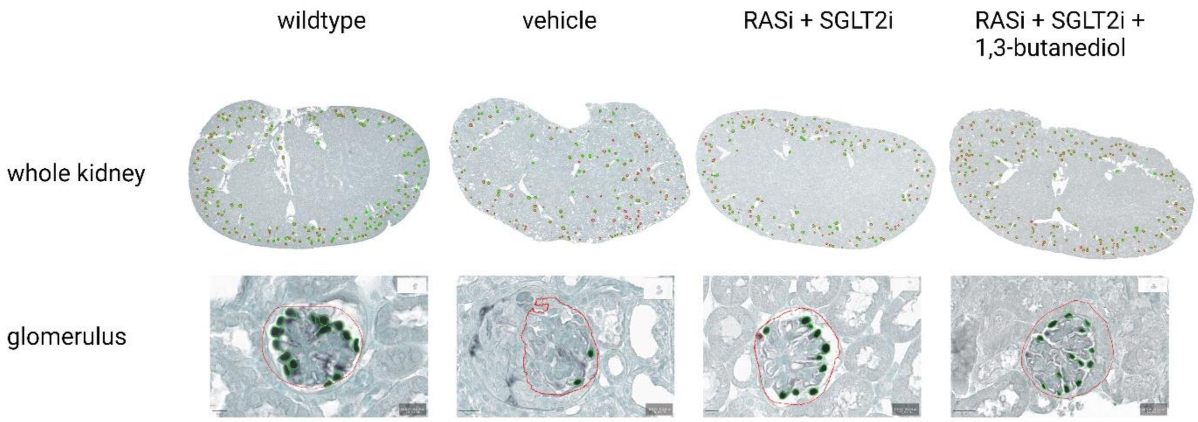

b

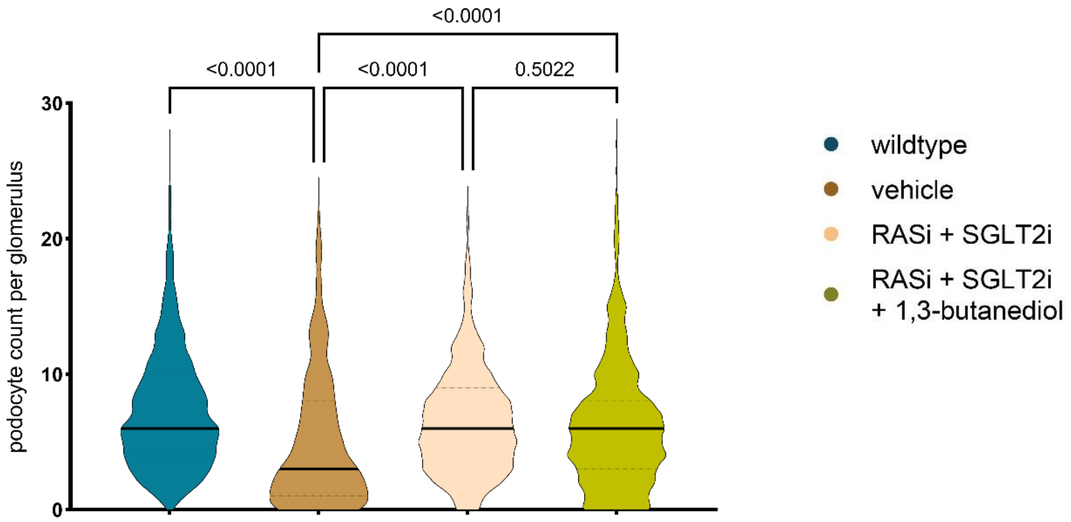

Supplementary figure 5: p57 staining

(a) Deep learning-based annotation of podocytes and glomeruli in representative whole kidney slides and in one representative glomerulus. (b) Podocyte count per glomerulus in different treatment groups. Testing for significance was carried out via ANOVA and Turkey´s multiple comparison test. The reference bar within each picture represents a length of 10µm. All quantitative data are means ± SD. A *P* value of <0.05 was considered to indicate statistical significance

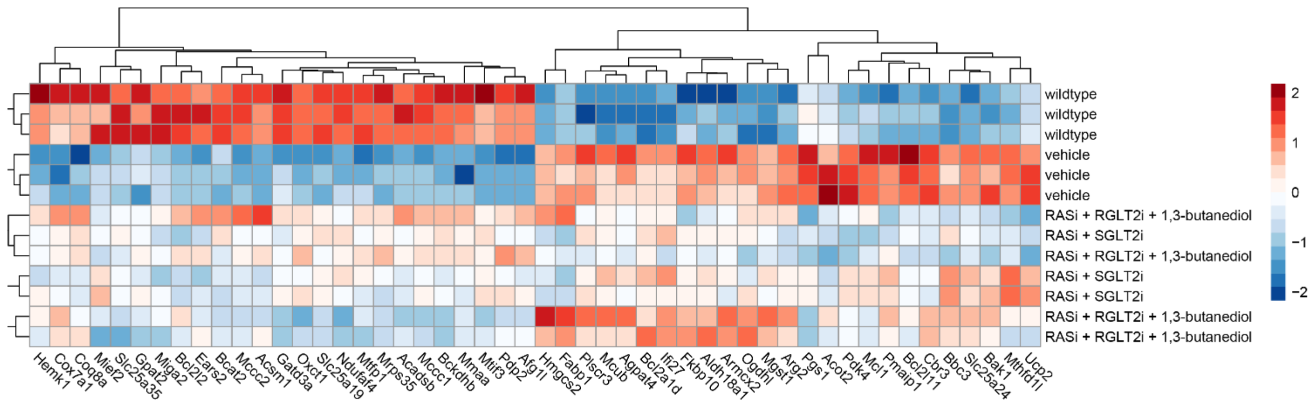

Supplementary figure 6: RNAseq for mitochondrial genes

(a) The heatmap presents the biological replicates of the top mitochondrial genes with differing expression levels between wildtype mice and Alport mice without, or with RASi + SGLT2i or RASi + SGLT2i + 1,3-butanediol treatment. A heatmap and dendrogram were used to display the differentially expressed genes' z-scores of normalized counts.

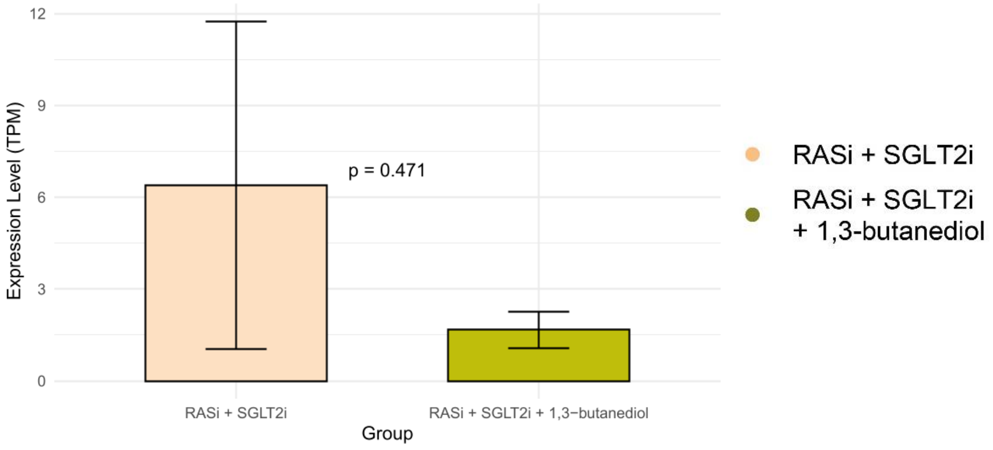

Supplementary figure 7: NLRP3 gene expression

(a) The column plot presents the TMP values (gene expression counts normalized across samples) for Alport mice with RASi + SGLT2i or RASi + SGLT2i + 1,3-butanediol treatment. Testing for significance was carried out by a Welch two sample t test. All quantitative data are means ± SD. A *P* value of <0.05 was considered to indicate statistical significance.

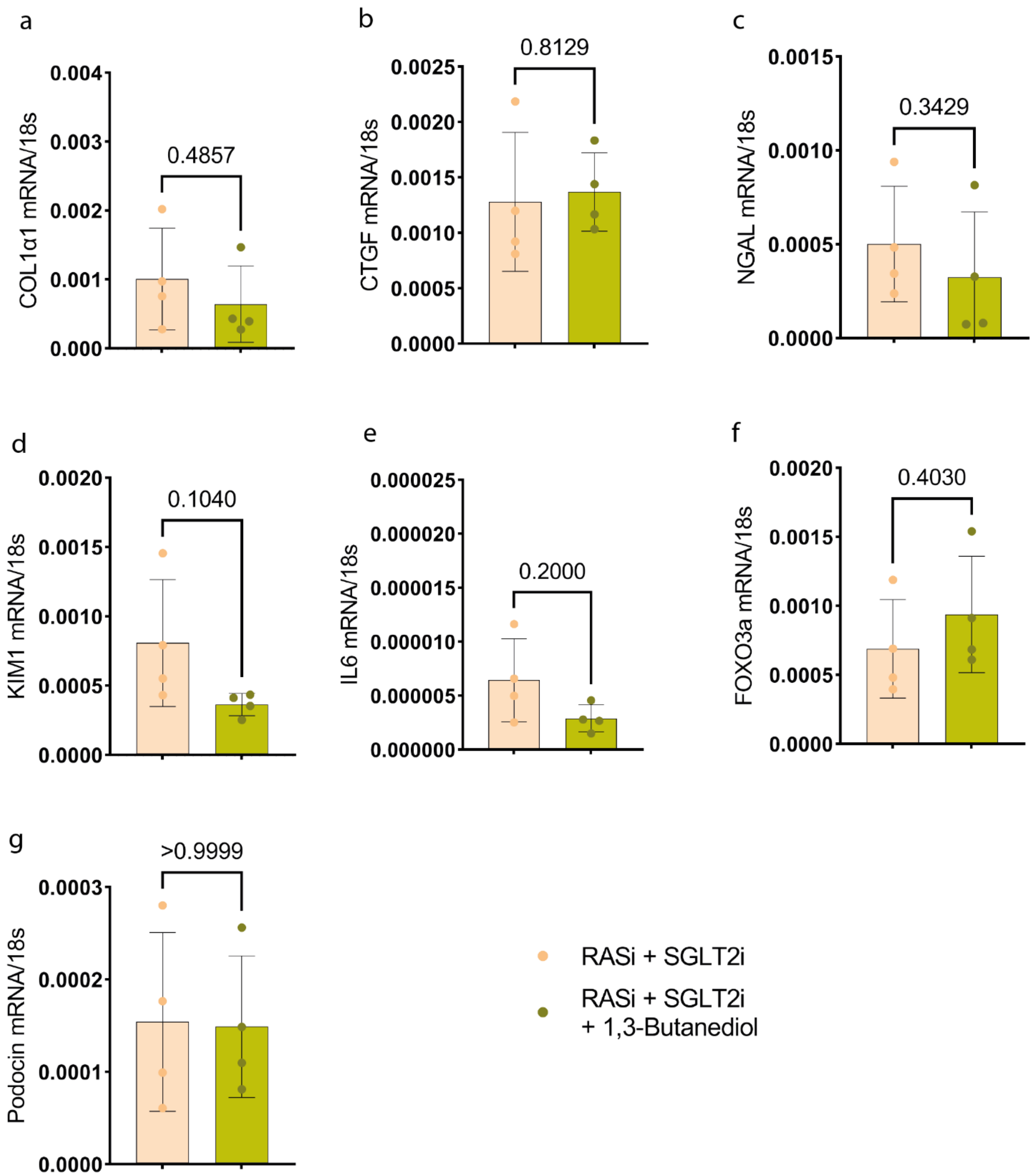

Supplementary figure 8: qPCR

Gene expression was analysed by RT-qPCR for fibrosis makers (a) *Col1a1* (b) *CTGF,* kidney injury makers (c) *NGAL* (d) *KIM1*, inflammation markers (e) *IL6* (f) *FOXO3a* and podocyte maker (g) *Podocin.* Testing for significance was carried out by either an unpaired t test for normally distributed data or a Mann-Whitney test for non-normally distributed data. All quantitative data are means ± SD. A *P* value of <0.05 was considered to indicate statistical significance.

Supplementary references

1. Taniguchi Y, Pippin JW, Hagmann H, et al. Both cyclin I and p35 are required for maximal survival benefit of cyclin-dependent kinase 5 in kidney podocytes. Am J Physiol Renal Physiol. May 1 2012;302(9):F1161-71. doi:10.1152/ajprenal.00614.2011
2. Bankhead P, Loughrey MB, Fernández JA, et al. QuPath: Open source software for digital pathology image analysis. Sci Rep. Dec 4 2017;7(1):16878. doi:10.1038/s41598-017-17204-5
3. Zhu Z, Rosenkranz KAT, Kusunoki Y, et al. Finerenone Added to RAS/SGLT2 Blockade for CKD in Alport Syndrome. Results of a Randomized Controlled Trial with Col4a3-/- Mice. J Am Soc Nephrol. Sep 1 2023;34(9):1513-1520. doi:10.1681/asn.0000000000000186
4. Zimmermann M, Klaus M, Wong MN, et al. Deep learning-based molecular morphometrics for kidney biopsies. JCI Insight. Apr 8 2021;6(7)doi:10.1172/jci.insight.144779
